# Magnetophoretic and spectral characterization of oxyhemoglobin and deoxyhemoglobin: Chemical versus enzymatic processes

**DOI:** 10.1371/journal.pone.0257061

**Published:** 2021-09-03

**Authors:** Mitchell R. H. Weigand, Jenifer Gómez-Pastora, James Kim, Matthew T. Kurek, Richard J. Hickey, David C. Irwin, Paul W. Buehler, Maciej Zborowski, Andre F. Palmer, Jeffrey J. Chalmers

**Affiliations:** 1 William G. Lowrie Department of Chemical and Biomolecular Engineering, The Ohio State University, Columbus, Ohio, United States of America; 2 Anschutz Medical Campus, University of Colorado, Aurora, Colorado, United States of America; 3 Department of Pathology, The University of Maryland School of Medicine, Baltimore, Maryland, United States of America; 4 Department of Biomedical Engineering, Cleveland Clinic, Cleveland, Ohio, United States of America; Bryant University, UNITED STATES

## Abstract

A new method for hemoglobin (Hb) deoxygenation, in suspension or within red blood cells (RBCs) is described using the commercial enzyme product, EC-Oxyrase®. The enzymatic deoxygenation method has several advantages over established deoxygenation methodologies, such as avoiding side reactions that produce methemoglobin (metHb), thus eliminating the need for an inert deoxygenation gas and airtight vessel, and facilitates easy re-oxygenation of Hb/RBCs by washing with a buffer that contains dissolved oxygen (DO). The UV-visible spectra of deoxyHb and metHb purified from human RBCs using three different preparation methods (sodium dithionite [to produce deoxyHb], sodium nitrite [to produce metHb], and EC-Oxyrase® [to produce deoxyHb]) show the high purity of deoxyHb prepared using EC-Oxyrase® (with little to no metHb or hemichrome production from side reactions). The oxyHb deoxygenation time course of EC-Oxyrase® follows first order reaction kinetics. The paramagnetic characteristics of intracellular Hb in RBCs were compared using Cell Tracking Velocimetry (CTV) for healthy and sickle cell disease (SCD) donors and oxygen equilibrium curves show that the function of healthy RBCs is unchanged after EC-Oxyrase® treatment. The results confirm that this enzymatic approach to deoxygenation produces pure deoxyHb, can be re-oxygenated easily, prepared aerobically and has similar paramagnetic mobility to existing methods of producing deoxyHb and metHb.

## Introduction

The magnetic properties of hemoglobin (Hb) and its derivative forms have been documented since the 1930’s [1, 2] and the magnetic separation of red blood cells (RBCs) using paramagnetic intracellular Hb is a continued pursuit in biomedical engineering. Many groups are investigating label-free methods to remove RBCs from whole blood by exploiting the diamagnetic properties of white blood cells and the paramagnetic properties of RBCs when the intracellular Hb is converted to the deoxygenated Hb (deoxyHb) or methemoglobin (metHb) state [3–18]. Another approach suggests it is possible to fractionate units of packed RBCs to positively separate RBCs with high magnetic susceptibility, containing the most Hb, before a transfusion to reduce the risks of transfusion associated circulatory overload, alloimmunization (particularly common with sickle cell patients) and increase post-transfusion RBC recovery above the 75% threshold for a “successful” transfusion [19–21]. We have developed, and continue to pursue, novel strategies to isolate RBCs and cell-free Hb from fresh and stored blood that aim to decrease the need for donated packed RBC units and improve patient outcomes.

Sickle cell disease (SCD) is a monogenic disorder where the patient is a heterozygous or homozygous carrier for the β^S^ allele. This mutation causes a single amino acid substitution from glutamic acid to valine in the β-globin chain of Hb and the resulting β6 substitution results in hydrophobic interactions with adjacent sickle cell Hb (HbS) molecules to yield HbS polymers. This polymerization process is triggered at low partial pressures of O_2_ (pO_2_) and is also dependent on intracellular HbS and HbF (fetal Hb) concentrations and pH. Studying how HbS is influenced by these parameters creates pathways for new diagnostic and therapeutic techniques. One specific need is a rapid test to treat a SCD “crisis”, in which rigid, sickled erythrocytes adhere to the endothelium and cause painful vaso-occlusion crises (VOC).

The primary treatment of SCD patients is to routinely receive exchange red blood cell exchange transfusions. During an exchange transfusion, allogenic normal RBCs are transfused to the SCD recipient while an apheresis device simultaneously removes the autologous RBCs from the transfusion recipient and returns plasma. Unfortunately, the removed blood contains a mixture of previously transfused normal cells and the patient’s SCD blood [22–24]. A useful improvement of this therapy would be to exploit a difference in magnetic susceptibilities (or other physical characteristics, such as size, density, deformability or response to inertial fluid forces) within a RBC population and discard only the HbS containing RBCs.

A common chemical reaction that yields metHb containing RBCs, is the addition of sodium nitrite to oxygenated Hb (oxyHb) containing RBCs in a simple, single step and has a conversion near 100% and is stable after several washing steps [9, 13, 18, 25, 26]. However, metHb containing RBCs is not a useful therapeutic, since metHb cannot bind to oxygen. Furthermore, the globin structure destabilizes upon metHb conversion to hemichrome when reacted with hydrogen peroxide (a byproduct of the reaction with nitrite) or diazine. MetHb can react with semiquinones to form oxyHb once again, but the formation of metHb is stable over a long period of time and is irreversible for practical uses such as paramagnetic RBCs separation with subsequent characterization of the RBCs [26]. Winterbourn demonstrated a reaction with superoxide to yield metHb, but this reaction is slow and simultaneously proceeds in the opposite direction [27, 28]. Di Iorio [29] and others prepare metHb using potassium ferricyanide but this method requires a second chemical reaction to yield oxyHb, as does sodium nitrite.

In contrast to placing a RBC in the met state chemically, there are two commonly reported ways to put a RBC in a deoxy state: chemically consuming the oxygen in the suspending buffer, or removing the oxygen from the buffer through gas stripping with a stripping gas. The common chemical method is the use of sodium dithionite [5, 15, 16, 30]. In an aqueous solution, sodium dithionite reacts with oxygen and water to produce sodium bisulfite and hydrogen peroxide. If used in dilute Hb solutions, hydrogen peroxide, and subsequently free radicals, become more abundant. In the solid phase, sodium dithionite will react with ambient oxygen to produce sulfite and thiosulfate, making weight measurement of the salt inaccurate. The actual amount dissolved into a sample must be determined from titration with a standard ferricyanide solution and following reduction at 420 nm (or measuring absorption at 314 nm, which is less accurate) [29, 30]. Lastly, dithionite has been shown to increase the 800 nm isosbestic point between oxyHb and deoxyHb due to side reactions [31]. The second isosbestic point, at 680 nm, absorbs much more weakly than at 800 nm. These points make sodium dithionite experimentally inaccurate and risky in clinical settings.

Deoxygenated RBCs can be achieved by bubbling N_2_, Ar, or another oxygen-free inert gas through the RBC solution or via application of a gentle vacuum [32, 33]. Moore et al. [17] used a hollow fiber deoxygenator to deoxygenate RBCs, but methods such as this that involve gas exchange must keep the system sealed from the atmosphere to properly deoxygenate the RBC solution. The oxygenation kinetics of deoxyHb containing RBCs and cell-free deoxyHb are discussed in Coin and Olson [34] and several other studies have described kinetic models and rate constants for other reactions involving Hb derivatives [25, 35, 36].

In contrast, and to the best of our knowledge, no reports exist on the use of the commercial enzyme product, EC-Oxyrase® (referred to as Oxyrase moving forward, not to be confused with the company name), to deoxygenate RBCs. In brief, Oxyrase refers to an enzyme system that allows radiation-damaged cells to divide, without entering or further damaging the cell. With addition of a proton donor, the enzyme consumes dissolved oxygen and converts it to water in media and deoxygenates the media without airtight containers or flushing the solution with inert gases [37]. The rate of dissolved oxygen removal is highly pH dependent, with a maximum activity at pH 8.4 and 55°C [38]. Other uses of Oxyrase include growing anaerobic cells, increasing cell growth in the log phase, increasing maximum titer and protecting certain biomolecules from reactions with oxygen [37, 39].

The active components of Oxyrase have been shown to repair damaged cells, accelerate cell growth and consume dissolved oxygen. These enzymes, which also originate from the cytoplasmic membrane fraction of E. Coli, have high reactivity with NADH and succinate [37]. These processes are due to active electron transport chain (ETC) complexes by which ATP is produced from NADH or succinate and H^+^ ions from within the cell. Mechanisms in eukaryotic and prokaryotic are similar, however they take place in different spatial domains. Eukaryotic ETC reactions take place across the inner membrane of the mitochondria, between the matrix and inner membrane space while still within the outer membrane of the cell. Prokaryotic ETC reactions takes place across the membrane, between the cytoplasm and outside of the cell. Five complexes (C_I_, C_II_, C_III_, C_IV_, C_V_) are involved. Briefly, C_I_ and C_II_ catalyze the reactant (NADH or succinate for C_I_ and C_II_, respectively) into ubiquinone (QH_2_) and transport the product to C_III_. C_III_ and C_IV_ are cytochromes, which contain active heme molecules (six for C_III_ and two for C_IV_). C_III_ transports 2H^+^ to C_IV_ via cytochrome c. Cytochrome c is a mobile electron carrier, while the transport chain from C_I_ or C_II_ to C_III_ occurs within a supercomplex of C_I_-C_III_-C_IV_ or C_II_-C_III_-C_IV_. C_IV_ reduces dissolved oxygen with the ions from cytochrome c to produce H_2_O [40–42].

This study evaluates the performance of Oxyrase to remove oxygen from intracellular Hb contained inside RBCs and cell-free Hb; resulting in changes in the magnetic susceptibility, and correspondingly when the Hb is inside RBC, the RBC magnetic susceptibility. The results from the use of an Eppendorf BioSpectrometer Basic, and the Winterbourn equation [27] and CTV [8, 18, 43–46], were used to experimentally quantify the deoxygenation of suspended Hb and Hb retained within RBCs, respectively. The motivation for this current study is to develop a convenient, easily reversible, nontoxic, single step deoxygenation of RBCs to magnetically separate RBC samples based on iron content for autologous re-transfusion.

## Materials and methods

### Human subjects

Blood samples were drawn from two healthy male (M1, M2) and female (F1, F2) donors with informed consents according to a protocol approved by the Institutional Review Board (IRB) of The Ohio State University (protocol number 38334). RBCs from SCD patients were provided by Dr. Irwin at the University of Colorado Anschutz Medical Campus as apheresis waste from three patients undergoing scheduled red blood cell exchange transfusions. These SCD blood samples contain a mix of normal donor RBCs and SCD patient RBCs. These samples were considered waste products and deidentified.

### Blood collection

These donors’ blood was collected into EDTA-coated vacutainers. SCD RBC samples were obtained from citrated solution. Samples were then diluted 1:100 in PBS, pH 7.4. After dilution, RBC concentration and size were measured in a Multisizer 4e Coulter Counter (Beckman Coulter).

### RBC and free Hb deoxygenation and oxidation

Oxygenated RBCs or cell-free oxyHb were converted to the deoxygenated state by adding Oxyrase (Oxyrase Inc.) with 60 wt.% sodium DL-lactate to maintain a pH suitable for a rapid deoxygenation rate. For comparison, deoxyRBCs or deoxyHb were obtained using 783 μM sodium dithionite (without using an inert gas), and 5 mM sodium nitrite was used for oxidation of Hb into the metHb form. Oxyrase and lactate were mixed by pipette and left in an aerobic environment for 30 minutes, and dissolved oxygen was confirmed to be < 1.0% using a ThermoFisher Orion 083005MD dissolved oxygen (DO) probe. Cell-free Hb and RBCs were added to PBS containing either Oxyrase/lactate, sodium dithionite, or sodium nitrite while exposed to the atmosphere. The manufacturer of Oxyrase defines 1 unit of EC-Oxyrase® as the “amount of Oxyrase that, under defined conditions, reduces dissolved oxygen at the rate of 1% per second” [47]. In this study, we do not report Oxyrase in units defined by the manufacturer but rather the volume fraction of Oxyrase in the sample. In a reaction to eliminate dissolved oxygen, Oxyrase acts as the enzyme for dissolved oxygen reduction to water in the presence of an appropriate hydrogen donor. In the present case, Oxyrase and lactate are mixed beforehand to prepare the anaerobic solution before oxyHb is added.

### Preparation of cell-free Hb

Cell-free Hb was prepared by lysing healthy RBCs in a -86°C freezer and thawing it in a bath of room temperature water. Cell lysate was centrifuged at 2,000 RCF for 5 minutes in an Eppendorf 5415c centrifuge. Spectrophotometry was performed by adding the supernatant to cuvettes containing the appropriate salt or enzyme in an Eppendorf BioSpectrometer Basic, while measurements were taken at chosen time intervals.

CTV was performed as previously described [8, 18, 43–46] to characterize deoxyRBCs and metRBCs. During this experiment, CTV samples were measured at a position within the magnet with a magnetic energy gradient (S_m_) value of 224.9 TA/mm^2^ (6.47 mm from the converging axis of the pole pieces). The Hb content on a cellular basis was calculated using CTV data and the method presented by Chalmers et al. [46]. Briefly, a force balance on RBCs (assuming Stokes drag and disc-shaped particles) accounts for the volumetric susceptibility of paramagnetic heme, diamagnetic globin and buffer, S_m_, viscosity, density, RBC trajectory and cell size to solve for intracellular Hb for each recorded particle on the CTV. During this analysis, the concentration of erythrocyte iron was calculated to be several orders of magnitude greater than the iron contained in the enzyme (presumably cytochrome c oxidase) and therefore the susceptibility of the RBCs and carrier fluid are unchanged by this addition.

### Spectrophotometry of Hb

For cell-free deoxyHb samples prepared with dithionite and Oxyrase, the percentage of oxyHb and deoxyHb was calculated using the method presented in [48]. A linear correlation of the percentage of oxygen saturation as a function of a ratio of the extinction coefficients ε_575_/ε_505_ was obtained by measuring oxygen-saturated Hb and a completely anaerobic sample of deoxyHb. Using equal Hb concentrations, the extinction coefficients in the Beer-Lambert law can be replaced with the absorbance measured via spectrometry. A control 100% oxyHb was diluted with PBS exposed to air because if left in an anaerobic buffer, deoxygenation would take place. Measurements were taken every 10 seconds after adding oxyHb to the deoxygenated buffer and continued until the absorbances at 505 and 575 nm remained steady for several consecutive measurements, which determines the 0% oxygenated point. OxyHb samples and those treated with sodium nitrite (suspected of also containing metHb and hemichrome) were evaluated using the equation reported by Winterbourn [27].

### Determination of oxygen equilibrium parameters

Oxygen equilibrium curves were measured using a Hemox Analyzer (TCS Scientific Corp) while maintaining the temperature at 37°C. For samples using Oxyrase, a temperature-controlled sample without Oxyrase and lactate was bubbled to 100% oxygenation using air, flushed out and refilled with a sample containing Oxyrase that was placed in a separate 37°C temperature bath and the measurements proceeded without bubbling nitrogen. During these experiments, the sample temperature deviated sightly (1.5°C to 3.0°C) from the setpoint.

## Results and discussion

### Reactions with free hemoglobin from healthy donors

**Fig 1:**
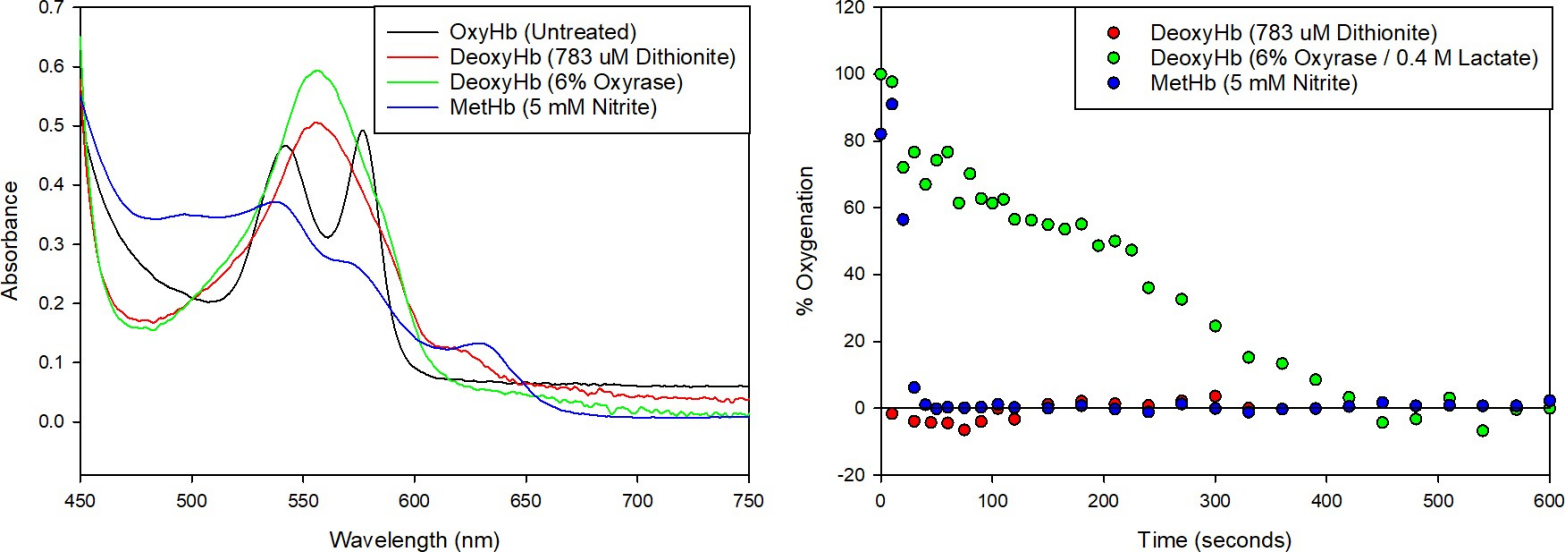
A. Spectra of oxyhemoglobin, deoxyhemoglobin and methemoglobin. The spectra of Hb is compared between an untreated, oxygenated sample (black) deoxygenated with 0.78 mM dithionite (red) with a secondary shoulder around 630 nm, suggesting metHb is also present. 6% Oxyrase and 0.4 M lactate (green) curve shows pure deoxyHb and the 5 mM nitrite (blue) curve shows pure metHb. B. OxyHb consumption over time between three Hb reactions. Free oxyHb is treated with 0.78 mM dithionite (red), 6% Oxyrase and 0.4 M lactate (green) and 5 mM nitrite (blue). The percentage of oxyHb is calculated over time using the Tsao method for deoxyHb (dithionite and Oxyrase) and the Winterbourn method (nitrite).

Publications studying deoxygenated Hb reacting with oxygen and several biomolecules including spinach ferredoxin, horse metmyoglobin, horse heart ferricytochrome with dithionite report reactions occurring on the order of milliseconds [49]. While using a basic spectrophotometer rather than a robust, airtight stopped-flow spectrophotometer, we qualitatively observed a very rapid deoxygenation of Hb, confirming literature observations. The spectra and relative oxygenation state over time for 13.4 ± 2.7 μM Hb for three different reactions are shown in Fig 1A and 1B, respectively. Fig 1A is presented as a normalized absorption intensity; spectra for dithionite treated Hb has a notable shoulder at 630 nm, consistent with the presence of metHb. Di Iorio [29] has reported that unstable dithionite produces H_2_O_2_, which can oxidize oxyHb into metHb [27]. In contrast, the reaction with Oxyrase shows no unwanted secondary peaks. As expected, the reaction in Fig 1B with dithionite is completed on the order of seconds, the reaction with sodium nitrite is on the order of 1 minute and the reaction with Oxyrase is completed on the order of 5–10 minutes. Overlaid spectra from deoxyHb treated with Oxyrase shows no significant change to the isosbestic point at 505 nm reported in [48].

**Fig 2:**
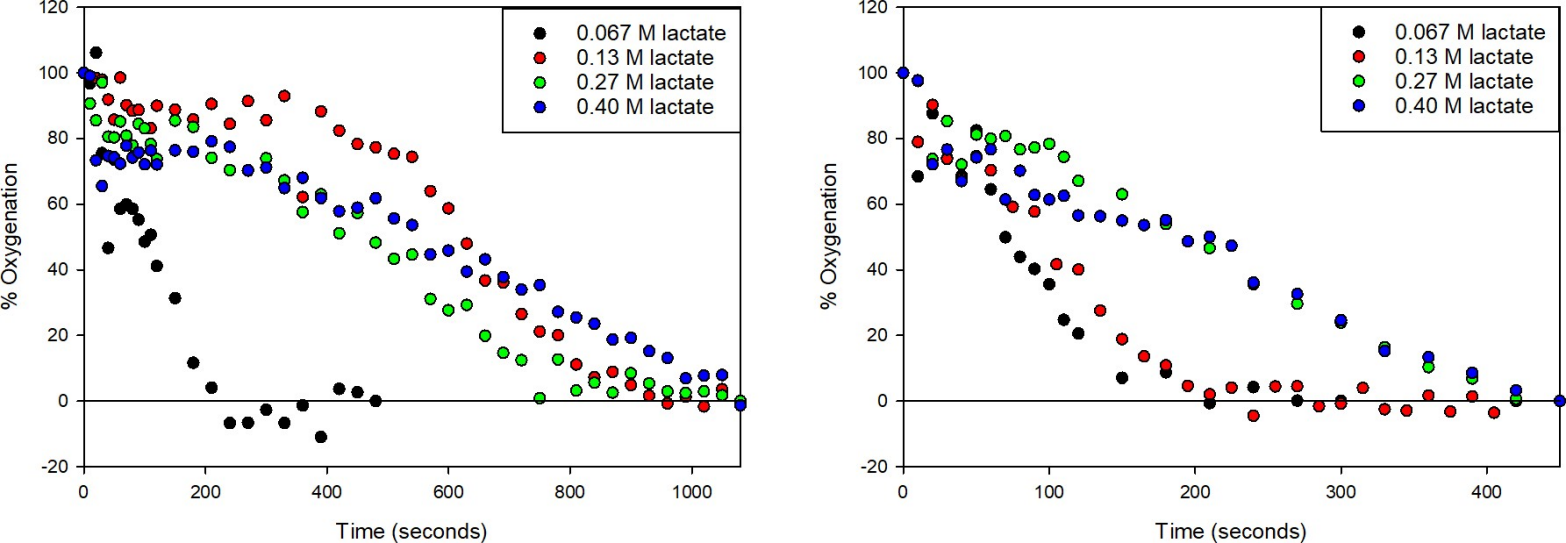
A. OxyHb consumption over time using 4% vol Oxyrase with different lactate concentrations. Hb is treated with 4% vol Oxyrase and lactate and the % oxyHb is calculated using the Tsao method for deoxyHb. B. OxyHb consumption over time using 6% vol Oxyrase with different lactate concentrations. Hb is treated with 6% vol Oxyrase and lactate and the % oxyHb is calculated using the Tsao method for deoxyHb.

**Fig 3:**
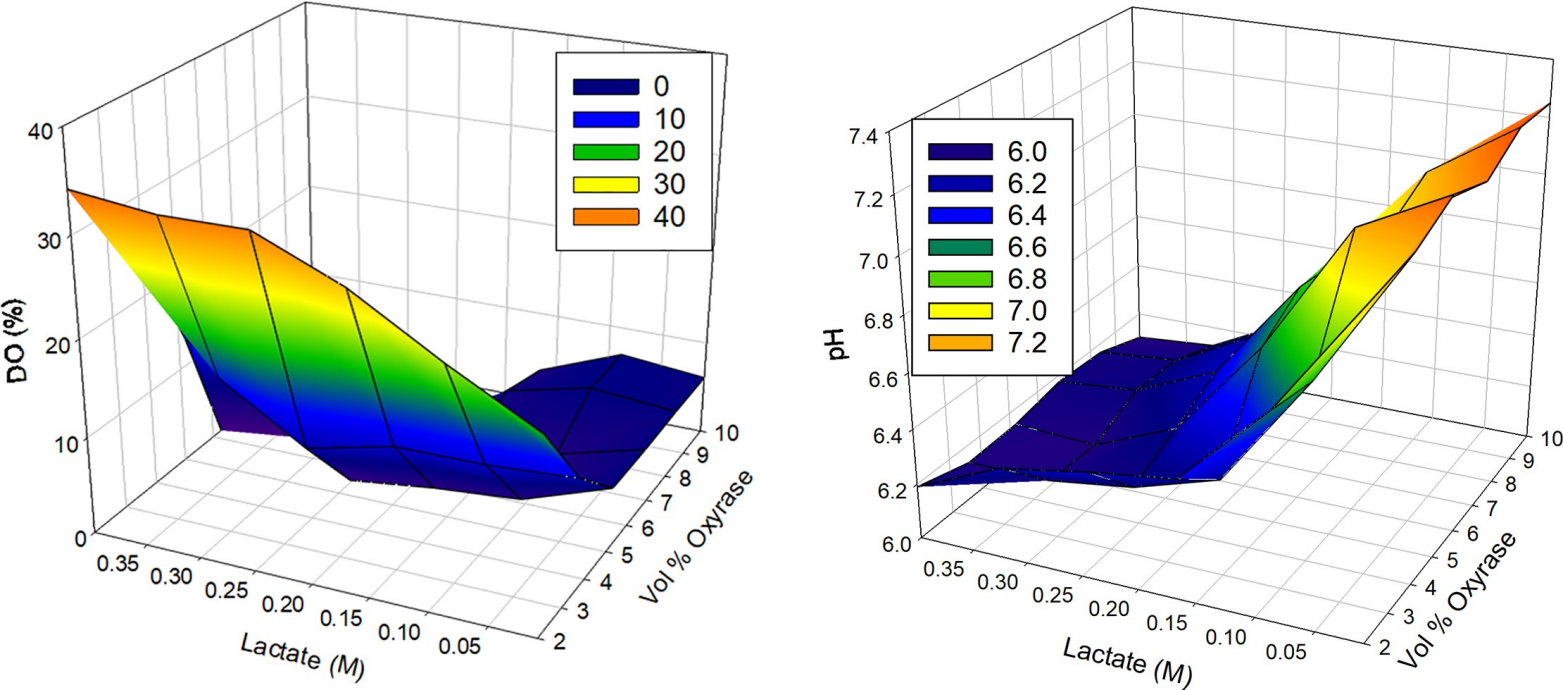
A. 3-dimensional map of DO mapped over lactate molarity and vol% Oxyrase. DO measurements are mapped as a function of added lactate and Oxyrase concentrations in PBS. B. 3-dimensional map of pH mapped over lactate molarity and vol% Oxyrase. pH measurements are mapped as a function of added lactate and Oxyrase concentrations in PBS.

Fig 2A and 2B present the percent oxygenation of Hb over time with varying lactate molarities and Oxyrase concentrations. Deoxygenation proceeds fastest with low sodium lactate and higher enzyme concentration. This observation is consistent with the measured DO in Fig 3A. At high lactate concentration, DO remains in the mixture and slow or incomplete conversion of oxyHb to deoxyHb may occur. Additionally, the amount of enzyme has a significant effect on DO. This result corroborates the effects of lactate molarity in Fig 2A and 2B. Fig 3B presents the solution pH as a function of lactate concentration and volume percent Oxyrase, and supports the conclusions that less lactate, higher pH (closer to the reported optimal pH for Oxyrase at 8.4) results in DO measurements that are near zero [39, 47]. Interestingly, pH increases with increasing enzyme in the buffer at low lactate concentrations but is unaffected by the amount of enzyme at high concentrations.

### Reactions with RBCs and CTV

**Fig 4:**
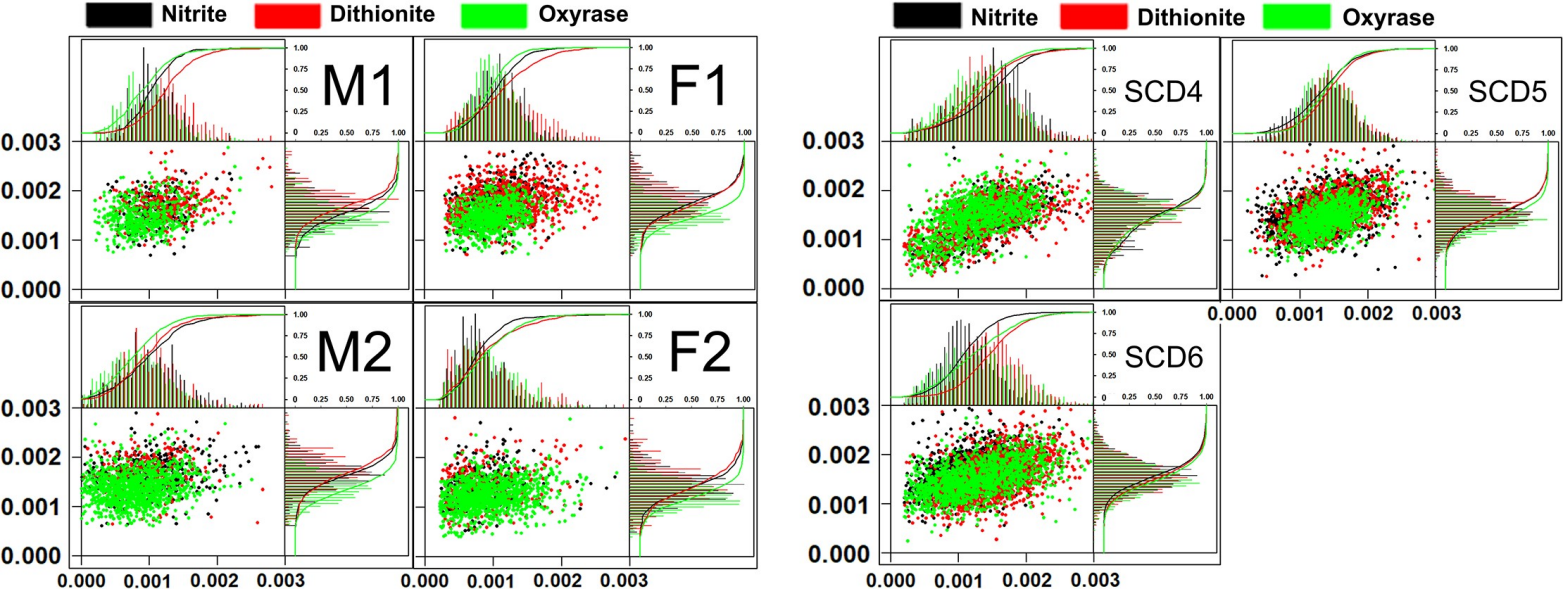
A. CTV data for two healthy male and two healthy female donor RBCs. Scatter plots with offset histograms and cumulative curves for the u_m_ (x-axis) and u_s_ (y-axis) for RBCs from four healthy donors that are treated with sodium nitrite (black), sodium dithionite (red) and Oxyrase (green). B. CTV data for three SCD donor RBCs of unknown gender. Scatter plots and histograms comparing three paramagnetic preparation methods on RBCs from three SCD patients of unknown gender are shown.

Fig 4 presents the magnetic velocity characteristics of paramagnetic RBCs from healthy donors. These graphs include the settling velocity, u_s_, versus the magnetic velocity, u_m_, scatter plot with appropriate histograms aligned along the side and top, respectively. The range in distribution presented here is consistent with what we have reported earlier in a study of 17 healthy donors [45]. Ongoing studies are investigating the significance of the sub-populations of cells that make up these distributions in healthy, anemic and SCD donors.

The histograms indicate that the distributions in both velocities nearly overlap for all three treatment methods across all four donors. However, due to increased viscosity of the lactate added to the Oxyrase-deoxygenated samples, the magnetic and settling velocities appear offset from the dithionite and nitrite samples, which can clearly be seen by following the smooth cumulative curves. This trend is more pronounced in the y-axis (settling velocity) than in the x-axis (magnetic velocity) and can be attributed to side reactions producing diamagnetic hemichrome (around 540 nm) due to over-oxidation in the case of nitrite. For example, the spectra of Hb in suspension (Fig 1A) indicates high amounts of hemichrome contaminating the metHb solution produced from exposure to nitrite. There are no other derivative forms of Hb present in these samples.

Fig 4B represents the same data for the three SCD donors. The scatter data for SCD RBCs are significantly wider than the u_m_-u_s_ scatter data for healthy RBCs. A large distribution in RBC size is suggestive of hypoxia and iron deficiency [50, 51], however this metric in our analysis does not differentiate these healthy and SCD donors using CTV (i.e., these samples, as stated above, are from the waste after a RBC exchange transfusion). Data in Fig 4A and 4B suggest similar distributions between u_s_ (which can be converted to cell size, assuming a constant cell density) histograms for healthy and SCD donors but shows that CTV is able to recognize a much wider u_m_ (analogous to pgHb/cell, potentially more clinically relevant) histogram for SCD donors compared to heathy ones. It is noteworthy that scatter plot data for SCD donors shows a correlation between u_m_ and u_s_, indicating that smaller/less dense cells have lower mobility, and therefore less Hb than larger/denser cells.

Although the gender and genotype of the SCD patients are unknown, scatter plot data in Fig 4B suggest that the SCD RBCs have a similar size to healthy RBCs, and not the rigid, elongated, polymerized homozygous HbSS RBCs.

**Fig 5:**
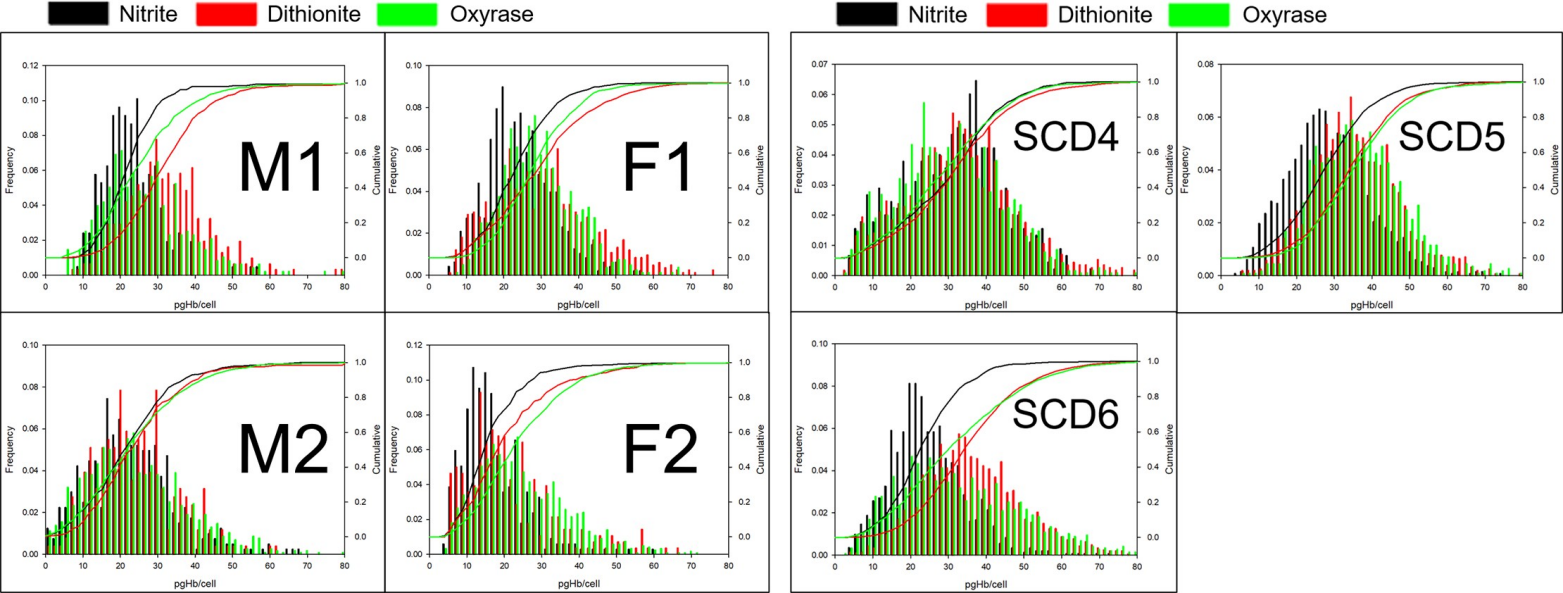
A. Picograms of Hb per cell for healthy donors. Using data from Fig 4 and the experimentally calculated Oxyrase viscosity from u_s_ comparisons within a donor, CTV data are converted to histograms showing the intracellular mass of Hb; sodium nitrite (black), sodium dithionite (red) and Oxyrase (green). B. Picograms of Hb per cell for SCD donors. Overlaid histograms and cumulative curves of three SCD patients compare intracellular Hb concentrations for three treatments.

Converting CTV data from Fig 4 to pgHb per cell [46], Fig 5A and 5B calculate the amount of Hb per cell for healthy and SCD donors, respectively. In this analysis, we account for the increased viscosity of Oxyrase and lactate in the reaction medium as well as u_s_ variations due to size. For each donor, the viscosity of the buffer is calculated to match the average u_s_ between the dithionite and nitrite samples was calculated, while using 0.9883 mPa*s for water at 20.5°C for dithionite and nitrite. The experimentally calculated viscosities for Oxyrase/lactate CTV samples for all donors are presented in Table 1 along with average and standard error of the donor’s iron status [52]. All calculated viscosities are within 15% of that of PBS and the added viscous transport limitation to the reaction is assumed to be negligible compared to pH dependence.

**Table 1:** Intracellular hemoglobin and experimental viscosities.

Donor	Gender	Status	Source	Average pgHb/Cell			pgHb/Cell SE			Number of Cells Tracked			Oxyrase Calculated Viscosity (Pa*s)
				Nitrite	Dithionite	Oxyrase	Nitrite	Dithionite	Oxyrase	Nitrite	Dithionite	Oxyrase	
M1	Unknown	Healthy	Donor	23.3	32.7	26.4	0.59	0.67	0.55	208	309	477	1.10E-03
M2	Unknown	Healthy	Donor	22.8	26.6	24.2	0.59	1.44	0.40	403	255	1156	1.18E-03
F1	Unknown	Healthy	Donor	24.4	30.5	29.1	0.41	0.47	0.35	479	830	802	1.15E-03
F2	Unknown	Healthy	Donor	17.4	21.9	24.9	0.61	0.76	0.36	336	280	1206	1.14E-03
SCD4	Unknown	Sickle Cell	Apheresis	31.2	32.9	30.4	0.62	0.43	0.41	449	1134	1153	9.87E-04
SCD5	Unknown	Sickle Cell	Apheresis	28.8	35.6	36.6	0.30	0.29	0.35	1317	1554	1123	1.06E-03
SCD6	Unknown	Sickle Cell	Apheresis	24.4	36.3	33.0	0.26	0.29	0.42	1441	2060	1478	1.03E-03
										Measured Viscosity (Pa*s)			1.78E-03
										Literature Viscosity (Pa*s)			1.12E-03

**Fig 6:**
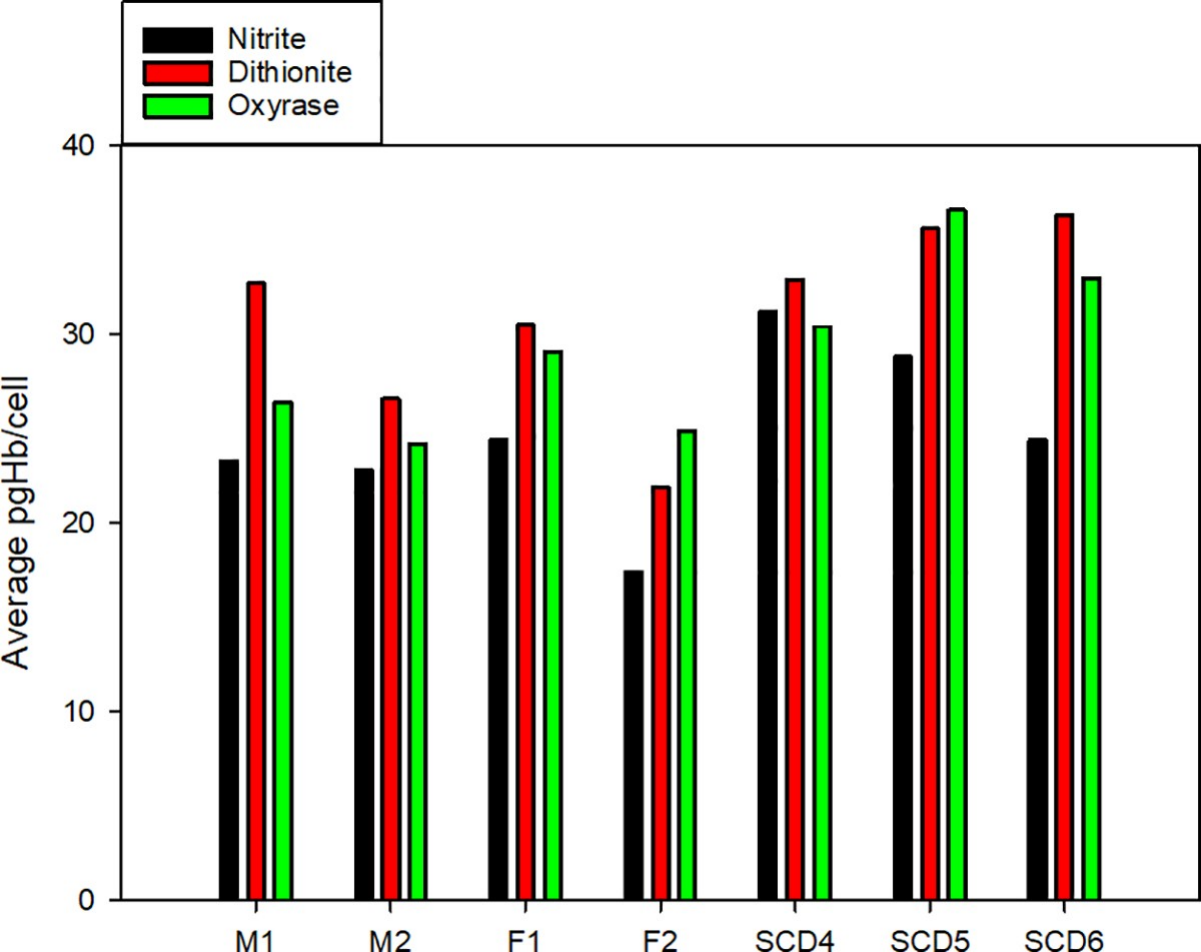
Average pgHb per cell for all donors. CTV data for all donors and conditions in Fig 5 are summarized in a bar graph.

Fig 6 compares average intracellular masses of Hb between preparation methods for each healthy and SCD donor. Interestingly, the average iron content between healthy and SCD donors is quite similar. SCD originating from the β-globin chain mutation polymerizes HbS and Fig 5A and 5B suggest that HbS-containing RBCs retain their intracellular Hb in this form. Lastly, it is suggested that CTV results for deoxygenated RBCs, induced by eliminating DO reflect the amount of iron that is available for oxygen binding and dissociation. If this is the case, magnetic mobility measured this way may reflect overall cell health and performance better than total iron (which may be influenced by chemical reactions), or total Hb test (intracellular and free Hb).

**Fig 7:**
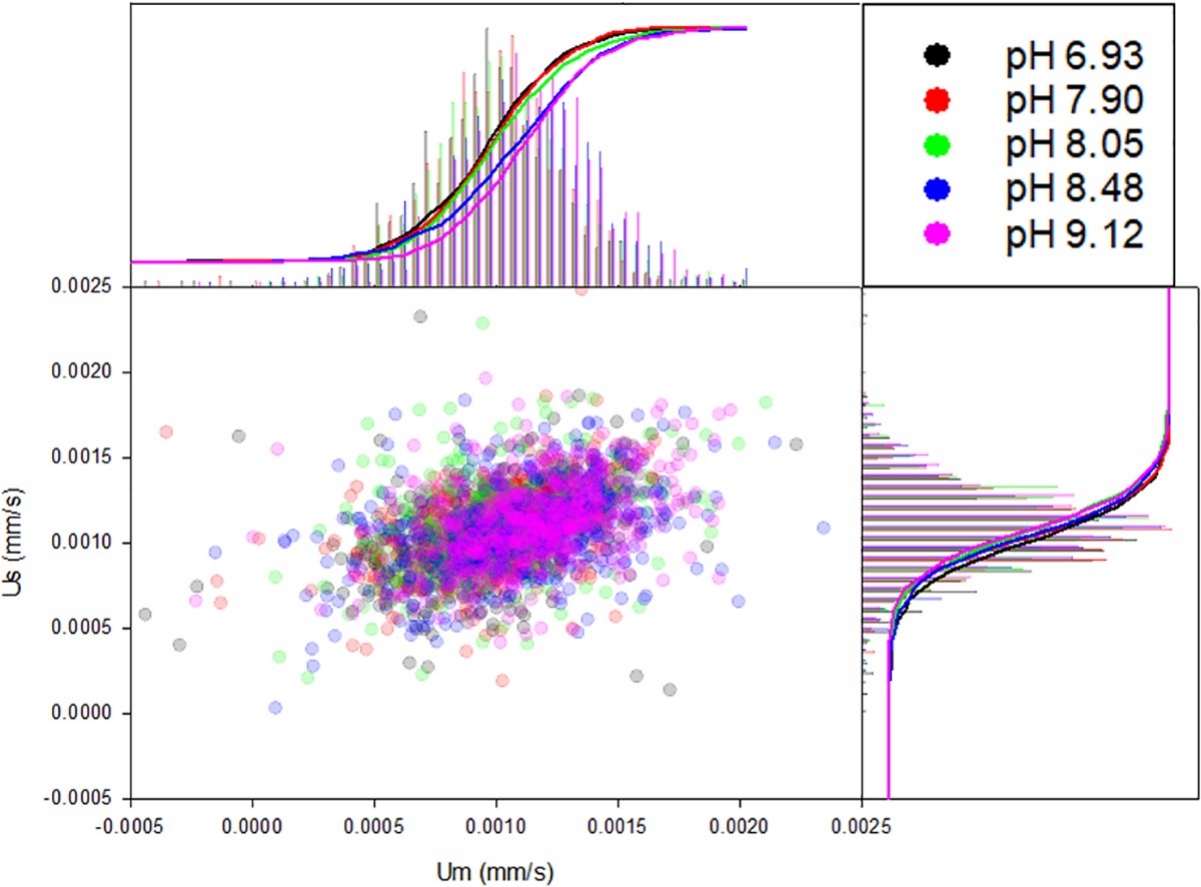
CTV data with AS-3 buffer pH gradient. A sample from donor F1 was measured using AS-3 as the RBC storage solution at five different pH values. Serial additions of NaOH increase pH and decrease viscosity. Converting to pgHb/cell reveals an average of 26.7 (±1.1 SD) pgHb/cell between the buffers.

The deoxygenation enzyme is active at high pH and subsequent experiments reveal improved methods to deoxygenate RBC buffer without significantly altering the viscosity. Most notably, the RBC storage solution AS-3 (consisting of salts, dextrose, adenine and ctiric acid to preserve the membrane, pH 5.8) with NaOH (added until the desired pH is achieved) in the presence of Oxyrase can completely remove DO in 15 minutes. AS-3 was chosen due to its RBC preservation properties and we see that Oxyrase is able to consume H^+^ while NaOH maintains the desired basic pH. Fig 7 shows CTV data from donor F1 (on a different date) with AS-3 as the RBC storage solution at five different pH values. Cumulative curves reveal that buffers with high pH have greater magnetic and settling velocities than AS-3 with low pH. This result is due to NaOH diluting the AS-3 and decreasing viscosity while equilibrium DO remains at zero. Using the same analysis to correct for viscosity, the pgHb/cell between the five buffers closely match (average 26.7 ± 1.1 SD pgHb/cell).

**Fig 8:**
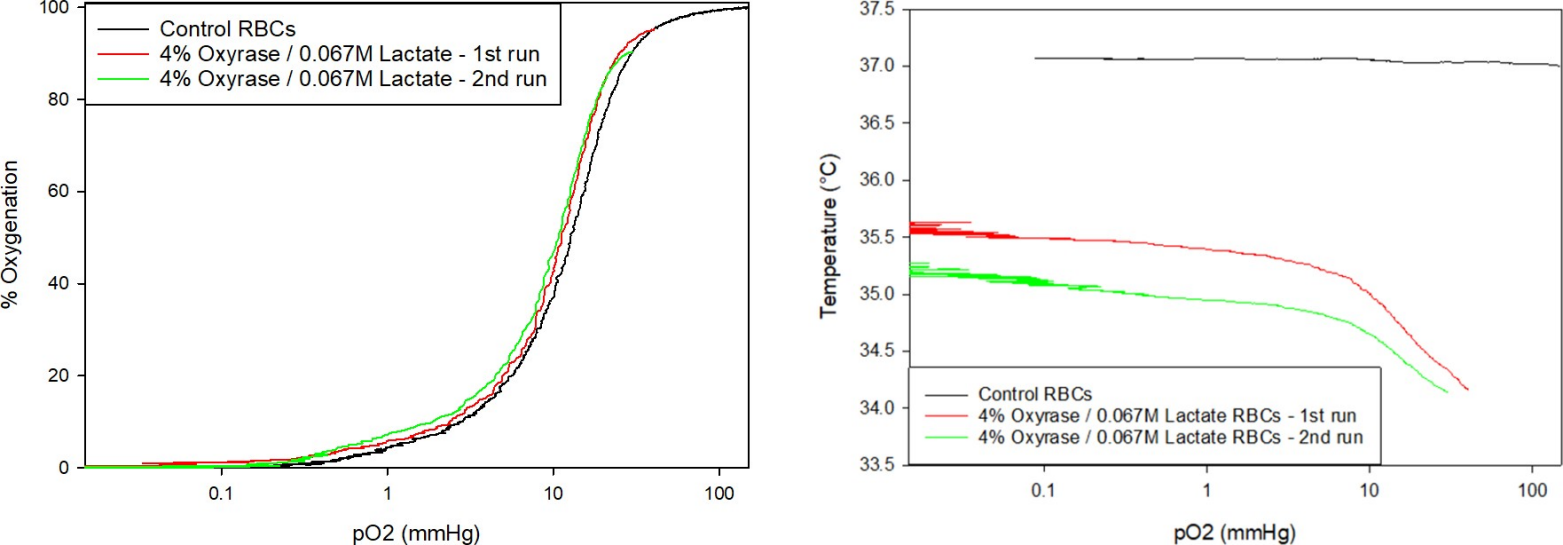
A. Oxygen equilibrium curves for healthy Hb. The control sample was produced by bubbling nitrogen gas through the sample, while the experimental sample was deoxygenated using Oxyrase. B. Temperature data for oxygen equilibrium curves. The left shift for the enzyme-treated Hb in Fig 8A is consistent with decreased temperature, due to the absence of temperature control during the enzymatic reaction.

Fig 8A presents the oxygen equilibrium curves of healthy donor Hb deoxygenated with N_2_ and two samples deoxygenated with Oxyrase (all samples 2 mg Hb/mL). The Hemox-Analyzer was used per protocol to obtain a normal curve at 37°C. The other two curves were obtained by adding oxyHb to a completely deoxygenated buffer (maintained at 37°C by a separate water bath) and quickly filling the measurement chamber while keeping the other settings consistent. The characterization functionality of the Hemox-Analyzer was used without the temperature-controlled deoxygenation. The first point recorded for these data is the point of maximum oxygenation, which was matched with the control sample in Fig 8A. The three curves are nearly identical, suggesting that the ability of Oxyrase-deoxygenated RBCs to bind to oxygen is identical to unaltered RBCs. The samples with Oxyrase and lactate have a small leftward shift from the control, which is consistent with that of a sample at lower temperature due to unconventional instrument use, shown in Fig 8B. These results suggest that the cells maintain their oxygen binding properties after exposure to Oxyrase. Additionally, Chang et al. report that murine RBCs stored in AS-3 at a basic pH have significantly less lipid peroxidation, phosphatidylserine expression, microvescicle production and hemolysis during storage as well as increased lactate concentrations (suggesting more metabolic activity) [53]. Therefore, enzymatic facilitated deoxygenation of RBCs in AS-3 may be a viable way to maintain low DO during a pre-transfusion enrichment while preserving cells’ metabolic, structural and reversible oxygen binding features.

## Conclusions

This study reports an enzymatic approach to facilitate Hb deoxygenation that is reversible, produces pure deoxyHb and reproducibly paramagnetic RBCs and can be done so in ambient conditions without need for an airtight chamber or sparging gas. Spectrometry and CTV results confirm that RBCs from healthy and SCD donors have similar paramagnetic behavior compared to established RBC treatments and can be fully deoxygenated in several minutes. Additionally, this enzymatic treatment does not affect the oxygen equilibrium properties of RBCs, making it a candidate for use in clinical translation The enzyme system is effective in a pH-controlled RBC storage solution, such as AS-3, that preserves membrane integrity and metabolic activity.

The effects on RBC morphology, metabolism and oxygen affinity are still unknown after prolonged exposure to Oxyrase (such as the 6 week storage life of donated RBCs) and should be observed before used in clinical settings. Additionally, experiments on pre-apheresis SCD blood should be performed to better understand the magnetic signature of patient blood without the healthy donor’s cells present. Lastly, a more detailed investigation of the kinetics and how the osmolarity (amount of buffering), pH, and presence of other chemicals would reveal more about the enzyme system and allow faster removal of DO.
